# Epicardial Coronary Arteries in Khat Chewers Presenting with Myocardial Infarction

**DOI:** 10.1155/2013/857019

**Published:** 2013-10-03

**Authors:** Ahmed Al-Motarreb, Adel Shabana, Ayman El-Menyar

**Affiliations:** ^1^Department of Cardiology, Faculty of Medicine, Sana'a University, Sana'a 13078, Yemen; ^2^Department of Cardiology, Heart Hospital, Hamad Medical Corporation, 3050 Doha, Qatar; ^3^Department of Cardiology, Faculty of Medicine, Ain Shams University, Cairo 11361, Egypt; ^4^Clinical Medicine, Weill Cornell Medical College, 24144 Doha, Qatar; ^5^Cardiology Unit, Internal Medicine, Ahmed Maher Teaching Hospital, Cairo, Egypt; ^6^Clinical Research, Trauma Surgery Section, Hamad Medical Corporation, 3050 Doha, Qatar

## Abstract

*Background*. Khat chewing is a common habit in Yemen despite increased evidence of its negative impact on the cardiovascular system. *Aims*. We aimed to study the epicardial coronary arteries in khat chewers presenting with myocardial infarction (AMI). *Materials and Methods*. A descriptive, cross-sectional study was conducted between November 2008 and May 2009 in Yemen. AMI patients who underwent coronary angiogram were enrolled and divided into groups (gp): gp1 (diabetic and khat chewers), gp2 (khat chewers and nondiabetic), and gp3 (diabetic and non-khat users). *Results*. Of 347 AMI patients 63%, 21%, and 16% were in gp 2, 3, and 1, respectively. Khat chewers were younger in comparison to non-khat users. Group 3 patients were more likely to have multivessel disease, severe left anterior descending (LAD), right coronary artery (RCA) stenosis and total RCA, and left circumflex (Lcx) occlusion compared to other groups. Group 1 patients were more likely to have total LAD occlusion and severe Lcx lesions. In multivariate analysis, age, diabetes mellitus, and smoking were significant independent predictors for significant coronary artery lesions; however, khat chewing did not show such association. *Conclusions*. Coronary spasm is the main mechanism of AMI in khat chewers. The impact of our finding for risk stratification and management warrants further studies.

## 1. Introduction

**Figure 1:**
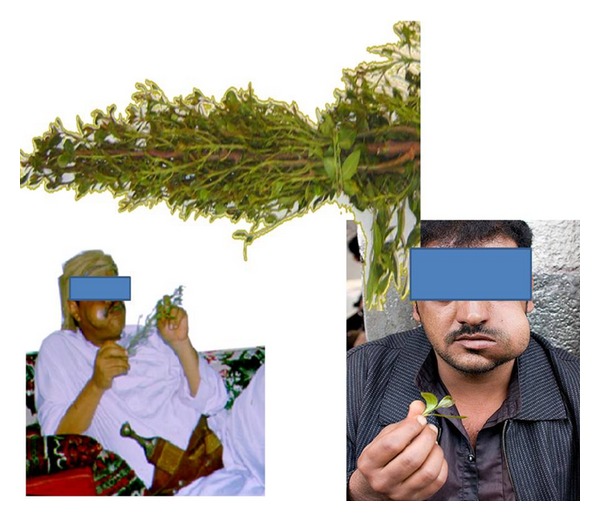
Khat tree and how khat users chew and keep it for few hours in their mouth cavity.

Khat (*Catha edulis*) is a flowering plant native to the Horn of Africa and the Arabian Peninsula [1]. In countries from these areas, “khat parties” have been accepted for decades as part of formal social customs [2]. Khat was initially thought to be a nonsignificant problem to Western populations; however, international delivery systems and immigration of khat chewers contributed to its large-scale distribution. Moreover, new synthetic forms are being developed, including Hagigat, Rakefet, Mephedrone, and Graba [3]. Users chew this stimulant habitually in groups, for its euphoric effects and as a recreational drug. Chewing khat is both a social and a culture-based activity. It is said to enhance social interaction, playing a role in ceremonies such as weddings [1, 2, 4, 5]. In Yemen, there are about 44 different types of khat from different geographic areas of the country. Its taste varies from one kind to another and depends on the tannic acid content. Khat leaves have an astringent taste and have an aromatic odor. The young leaves are slightly sweet [6, 7] (Figure 1).

In 1980, the World Health Organization (WHO) classified khat as a drug of abuse that can produce mild to moderate degree of psychological dependence (less than tobacco or alcohol) [8]. Khat has been reviewed by the WHO Expert Committee on Drug Dependence (ECDD) several times; the most recent occasion was at the 34th meeting of ECDD in 2006. However, Catha edulis remains outside international control which does not consider khat to be seriously addictive [8, 9].

Khat contains several components, mainly a monoamine alkaloid called cathinone, which is an amphetamine-like stimulant that is responsible for most effects of khat, including excitement, loss of appetite, and euphoria [10, 11].

Cathinone and other ingredients of khat are structurally and functionally related to noradrenaline, and ecstasy (3,4-methylenedioxymethamphetamine) [10, 11]. In each session (party), about 100–200 mg of khat is consumed by a person. Such an amount is equivalent to an oral dose of 5 mg of amphetamine [1, 12]. Several substances such as amphetamine, ecstasy, ephedrine, and epinephrine have been reported to cause coronary vasoconstriction [13, 14]. The First and Second Gulf Registries of Acute Coronary Events (Gulf RACE-1 & 2) from 6 Middle Eastern countries including Yemen have recently shown that khat chewing is associated with worse outcome in patients with acute coronary syndrome (ACS), compared with non-khat users who are mainly from different Gulf populations [1, 3].

This study aims to assess, for the first time in humans, the detailed anatomical and morphologic changes of the coronary vessels in patients presenting with acute myocardial infarction (AMI) who are khat chewers compared to those who are not. Moreover, as it has been shown in several studies, diabetic patients may have diffuse or extensive degree of coronary artery disease (CAD) [15–20]. We analyzed the coronary morphology among khat chewers, taking the presence of diabetes mellitus into consideration as a control group.

## 2. Methods

The method of this study was descriptive, cross-sectional*‚* hospital-based. Patients who had been referred for coronary angiogram were enrolled during 6-month time from November 15, 2008, to May 15, 2009. Doctors interviewed all patients, and a predesigned questionnaire was filled. The time of interviewing the patients was just before the catheterization procedure. The study procedures were clearly explained, and consents were obtained and approved. Data were collected from patients who presented with AMI and underwent coronary angiographic study in the cardiac center, Al-Thawrah Hospital, Yemen. Patients were divided into 3 groups: group 1 (diabetic and khat chewers), group 2 (khat chewers and nondiabetic), and group 3 (diabetic and non-khat chewers). Data including demographics, clinical presentations, and coronary angiographic findings were analyzed and compared among the 3 groups.

### 2.1. Statistical Analysis

Patients' characteristics were presented as proportions, medians, or mean ±  SD, as appropriate. Chi-square (*χ* ^2^) test was used to compare categorical variables, and one-way ANOVA was performed for continuous variables. To examine the predictors for the presence of significant coronary artery disease (anatomically significant coronary artery disease was defined as a luminal stenosis greater than or equal to 50% in one or more epicardial arteries), multivariate logistic regression analysis was conducted after adjusting the significant relevant variables. Adjusted odds ratio (OR), 95% confidence interval (CI), and *P* values were reported for significant predictors. All *P* values were the results of 2-tailed tests, and values <0.05 were considered significant. Statistical Package for Social Sciences (SPSS) version 19.0 has been used for the analysis.

## 3. Results

Of 347 AMI patients, 55 (16%) were both khat chewers and diabetic (group 1), while 219 (63%) were khat chewers and nondiabetic (group 2) and 73 (21%) were diabetic and non-khat chewers (group 3). Khat chewers (both groups 1 and 2) represented 79% of the study population and were generally 5 years younger at presentation. Male-to-female ratio was nearly 3 : 1.  Table 1 shows the demographic, clinical presentation and coronary morphology in patients with acute myocardial infarction.

Khat chewers were more likely to be smokers and less to have hypertension in comparison to nonchewers. There was no significant difference in the location of MI in the 3 groups; however, anterior MI was the most prevalent in all groups.

### 3.1. Coronary Angiography

All patients in the study underwent diagnostic coronary angiography during the index admission. Group 2 (khat chewers only) patients were more likely to have non significant lesions (58%) including normal coronary arteries (44%) in comparison to the other two groups who were diabetic as well. Group 3 patients were more likely to have two- or three-vessel disease and more likely to have severe left anterior descending coronary artery (LAD) occlusion (70–95% occlusion), severe right coronary artery (RCA) stenosis (>70% occlusion) and total RCA, and left circumflex (Lcx) occlusion, compared to group 1 and 2 patients.

Group 1 patients were more likely to have LAD total occlusion, severe Lcx lesions, and first obtuse marginal (OM1) significant lesions (>70% occlusion) when compared to other groups.

Table 2 shows the multivariate regression analysis for predictors for significant coronary artery stenosis. In multivariate logistic regression analysis to demonstrate factors associated with significant CAD lesions, age, presence of diabetes mellitus (DM), and smoking were significant predictors for the severity of CAD lesions; however, khat chewing did not show such association.

## 4. Discussion

There are no published data describe the angiographic details of the epicardial coronary arteries in khat chewers presenting with MI. The present study aims to correlate khat chewing with CAD anatomy, taking in consideration the effect of DM on coronary morphology as a control group.

Among Yemeni patients with ACS, khat chewers represent 79% of the current study population. This percentage is nearly similar to previous reports from the same area [3, 7]; such prevalence may be due to the socially acceptable use of this habit during the “khat parties” that are common in Yemen [3].

In our study, khat chewers, especially nondiabetic, were younger in age than non-khat chewers. This is different from previous studies that found khat chewers older than control groups. However, the mean age of khat chewers was also different. This finding is consistent with previous study which showed that the age of khat chewers was younger than non-chewers in AMI patients [21]. However, another study showed that the mean age of khat chewers was older than the control group among ACS patients including unstable angina and non-ST elevation AMI [3].

In the current study, coronary morphology was significantly different in group II than in the other two groups. It seems that non-diabetic khat chewers were more likely to have normal coronaries or non-significant lesions rather than significant CAD. We analyzed data for khat chewers based on the presence or absence of DM as it is well known from previous reports that diabetes is associated with significant diffuse and extensive CAD [15–20]. Khat chewing increases the likelihood of having normal or non-significant coronary arteries in AMI patients in comparison to those who have DM.

Our data are concordant with previous analysis of the GULF RACE 2 study, although the overall use of coronary angiography was low. In GULF-RACE 2, 32 of the total 172 khat chewers (18.3%) who had coronary angiography had normal coronary arteries or nonsignificant lesions, and the remaining patients had evidence of significant coronary artery stenosis [1]. Our previous work demonstrated that in animal models, cathinone, “the main gradient of khat leaves,” produces coronary and aortic vasoconstriction; this effect was proved by inducing vasoconstriction in porcine left anterior descending coronary artery [22, 23]. Other clinical data showed that although the chewers were hot and sweet, their hands and feet were cold which indicates the vasoconstrictive effect of khat on the peripheral circulation. The mechanism of acute MI mechanism in khat chewers could be due to coronary vasospasm in addition to other factors such as platelet aggregation produced by excessive catecholamine release and acceleration of the atherosclerosis process.

**Figure 2:**
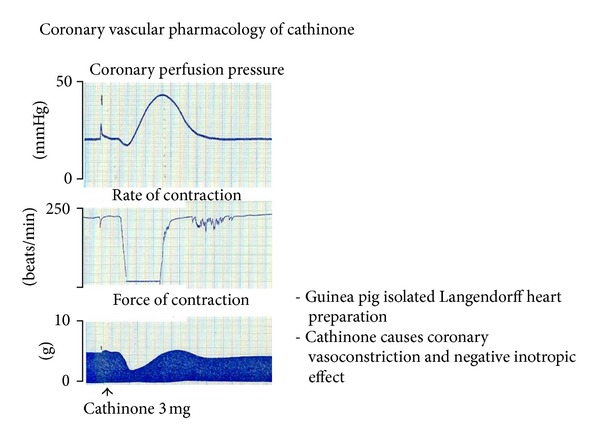
The vascular effect of cathinone in pigs; a courtesy from Dr. Ahmed Al-Motarreb.

Our previous data support the postulated mechanisms by which khat can induce AMI; Al-Motarreb and Broadley [22] reported that cathinone infusion induced marked vasoconstriction of the coronary vasculature in isolated guinea pig hearts (Figure 2). In another study, such vasoconstriction effect was not blocked by the alpha1-adrenoceptor antagonist prazocin or by the neuronal uptake inhibitor cocaine [23].

Khat-induced coronary spasm is further supported by the fact that compounds similar to cathinone, like amphetamine, cocaine, and ecstasy, have been shown to induce coronary artery spasm and precipitate AMI [13, 24–28]. Khat is a natural amphetamine [11]; therefore, we can assume that the two substances share the same mechanism for inducing coronary vasospasm and AMI.

In addition to this theory, cathinone could produce myocardial oxygen demand-supply mismatch, through increasing heart rate and blood pressure, especially in the first few hours after khat parties [21, 29–31].

Moreover, several mechanisms have been postulated, mainly from studies on similar compounds as amphetamine and cocaine, to explain the association of khat chewing with cardiovascular complications including hypercoagulable state, catecholamine-induced platelet aggregation, and premature coronary atherosclerosis [3, 32–34].

In previous studies in our area, khat chewers had significantly higher mortality rates compared with non-khat chewers (8.7 versus 2.9%; *P* < 0.001). They were also more likely to develop cardiogenic shock and stroke [1, 3].

### 4.1. Study Limitations

Our data were collected from an observational study. Long-term follow-up and large sample sized study are needed to support our findings.

## 5. Conclusion

Coronary spasm is the main mechanism of AMI in khat chewers. The impact of our finding at the risk stratifications and management warrants further studies in the khat-chewing communities and among immigrants from these communities to Europe and USA.

## Figures and Tables

**Table 1:** Demographic, clinical presentation, and coronary morphology in patients with acute myocardial infarction.

	DM and khat	Khat and non-DM	DM and non-khat	*P* value*
Number (%)	55 (16%)	219 (63%)	73 (21%)	
Age (mean ± SD)	54 ± 10	52 ± 10	57 ± 9	0.001
Females %	13	12	30	0.001
Myocardial infarction %	86	80	77	0.46
Hypertension %	46	33	49	0.02
Current tobacco users %	49	48	26	0.001
Received thrombolytics %	15	12	19	0.38
Coronary angiographic findings				
Left main disease %	5.5	2.7	1.4	0.34
LAD 70%–95%	26	14	32	0.005
Total LAD occlusion %	15	11	12	0.005
1st diagonal (D1) > 70%	3.6	8.2	9.6	0.90
D2 > 70%	5.5	2.7	2.7	0.72
1st obtuse marginal (OM) > 70%	12.7	1.4	2.7	0.008
OM2 > 70%	7.3	4.1	6.8	0.49
LCx 70%–95%	26	9	15	0.02
LCx total occlusion %	0	2.7	4.1	0.02
RCA 70%–95%	12.7	12	26	0.006
RCA total occlusion %	5.5	6.8	8.2	0.006
PAD > 70%	1.8	2.3	2.7	0.71
Normal coronary %	29	44	19	0.001
1-vessel disease %	31	24	21	
2-vessel disease %	22	13	33	
3-vessel disease %	7	5	11	
Nonsignificant lesions %	40	58	36	0.001
Location of myocardial infarction (MI) %				
Anterior MI %	73	63	72	0.64
Lateral MI %	4.5	13	7.5	
Inferior MI %	11.4	16.3	13.2	

*P value comparing the 3 groups.

**Table 2:** Multivariate logistic regression analysis for predictors for the presence of significant coronary artery stenosis*.

Variable	Odd Ratio	95% confidence interval	*P* value
Khat chewing	0.84	0.580–1.221	0.36
Diabetes mellitus	2.07	1.367–3.165	0.001
Hypertension	1.18	0.824–1.714	0.35
Age	0.95	0.937–0.972	0.001
Gender	1.55	0.965–2.477	0.07
Tobacco	1.67	1.191–3.072	0.01

*Anatomically significant coronary artery disease was defined as a luminal stenosis greater than or equal to 50% in one or more epicardial arteries.
